# Efficacy and safety of non-fractional ablative carbon dioxide laser resurfacing for the treatment of rhinophyma – a retrospective cohort and questionnaires-based study

**DOI:** 10.1007/s10103-025-04442-7

**Published:** 2025-06-09

**Authors:** Yehonatan Noyman, Hadas Ofer Friedman, Moshe Lapidoth, Gaia Harris Rimon, Efrat Solomon-Cohen, Igor Snast, Mati Rozenblat, Daniel Mimouni, Assi Levi

**Affiliations:** 1 Laser Unit, Division of Dermatology, Rabin Medical Center, Petah Tikva, Israel; 2 Faculty of Medicine, Tel Aviv University, Tel Aviv, Israel; 3 Department of Dermatology, “Emek” Medical Center, Afula, Israel

**Keywords:** CO2 laser, Rosacea, Rhinophyma, Resurfacing

## Abstract

**Purpose:**

Phymatous rosacea is a chronic and disfiguring subtype of rosacea, mainly affecting the nose and leading to the development of rhinophyma. This condition manifests with erythema, enlarged pores and increased sebum secretion, progressing to textural alterations and nasal hypertrophy. Ablative CO_2_ laser resurfacing has emerged as a preferred approach, offering hemostatic control and favorable cosmetic outcomes. This case series presents our treatment experience with a non-fractional ablative CO_2_ laser resurfacing under local anesthesia for severe rhinophyma patients.

**Methods:**

A Retrospective case series of patients with severe rhinophyma treated with an ablative CO_2_ laser between December 2010 and March 2020 in our laser unit. Post-procedure aesthetic outcome was assessed by the treating physician 3 months following treatment and patients were asked to complete a long-term follow-up questionnaire.

**Results:**

Sixteen patients (15 males) were included, of which 13 patients (81%) had completed the questionnaire on an average of 15 months following treatment (range 2–24 months). Patient satisfaction following treatment was high, with an average satisfaction score of 7.9 out of 10 (range 4–10). Post-procedure aesthetic outcome was rated as very good or excellent in 13 patients (81%, with 75% or greater improvement). Among the 13 patients who completed the questionnaire, 11 (85%) indicated that they would recommend this treatment to others with a similar condition.

**Conclusion:**

Non-fractional, ablative CO_2_ laser resurfacing performed under local anesthesia, has proven to be a safe, effective and well-tolerated treatment for severe rhinophyma, yielding sustainable results and high satisfaction rate.

**Supplementary Information:**

The online version contains supplementary material available at 10.1007/s10103-025-04442-7.

## Clinical trial number

Not applicable.

**Supplementary Information:**

The online version contains supplementary material available at 10.1007/s10103-025-04442-7.

## Introduction

Phymatous rosacea is a chronic, inflammatory, progressive, and disfiguring subtype of rosacea that most commonly involves the nose in a manner known as rhinophyma (Greek for nose growth), yet may also affect other facial structures, such as the chin, forehead, ears and eyes [1]. It is more prevalent in Caucasians, usually in their fifth to seventh decade, and unlike other types of rosacea, it demonstrates a male predominancy [2]. 

Clinically, rhinophyma affects the lower two-thirds of the nose and manifests with erythema, telangiectasias, enlarged pores, and increased sebum secretions. These symptoms gradually progress to textural alteration with thickening and roughening, and eventually develops to diffuse or lobular nasal hypertrophy [3]. 

Owing to the disfiguring nature of the disease, it is associated with detrimental effects on well-being [4, 5]. 

The etiology of rhinophyma is not well understood, and while environmental factors such as alcohol consumption and demodex folliculorum infection have been suggested, their role remains unclear [6–8]. 

While early-stage rhinophyma can be treated medically with oral anti-inflammatory antibiotics or oral retinoids, which can sometimes halt its progression, debulking procedures remain the mainstay of treatment in most cases. These procedures utilize a variety of modalities, including excision, electrosurgery, cryosurgery, dermabrasion, and laser therapy [9]. 

Ablative, non-fractional carbon dioxide (CO_2_) lasers have gained their place as the laser therapy of choice for rhinophyma, due to their superior hemostasis, which allows for a “cleaner” surgical field resulting in excellent cosmetic outcomes [10]. 

The 10,600 nm CO_2_ laser targets water and vaporizes the phymatous tissue under clear visualization to the level of the sebaceous glands. This reduces scarring, facilitates recovery, and optimally preserves the aesthetic sub-units of the nose [10]. Reepithelization is evident as early as 6 days post-treatment and is usually complete within a month. Adverse events are generally minor and infrequent, with the most common being pain from local anesthesia and post-procedural erythema. Less common complications include scarring, dyspigmentation, and localized infection [11]. Recurrence is rare and patient satisfaction rates are high, leading to a positive psychological effect with improvement in reported well-being and self-confidence [12]. 

General anesthesia, though sometimes administered [13], could be avoided since local anesthesia with repeated injections of local anesthetic solution (1% or 2% lidocaine or 0.25% bupivacaine, with or without a 1:200,000 mixture of adrenaline) to the nasal bone-cartilage and nose-cheek junctions is well tolerated and creates a nasal nerve block. This technique has the advantages of reducing bleeding and post-operative pain [12, 14]. 

In this case-series we present our experience treating severe rhinophyma patients with a non-fractional ablative CO_2_ laser.

## Methods

### Setting

This is a retrospective case series of patients with rhinophyma treated with an ablative CO_2_ laser between December 2010 and March 2020 in the laser unit of a tertiary medical center.

The study was approved by the Ethics Committee of Rabin Medical Center in accordance with the guidelines of the Helsinki Committee (0299 - 19 RMC).

### Patients and treatment protocol

All patients were referred to our laser unit by a dermatologist, where they were diagnosed with severe rhinophyma, characterized by generalized hypertrophy and/or local lobular nodulation of the lower part of the nasal bridge, nasal tip or alas.

Prior to treatment, all patients received a detailed explanation regarding their diagnosis, the ablative CO_2_ laser procedure, potential adverse events and alternative management options. Patients signed an informed consent form and were photographed.

Treatment preparation included disinfection, administration of an injectable local anesthetic agent (1% lidocaine) and the coverage of patient’s eyes with protective goggles.

Hypertrophic tissue was ablated to the level of the sebaceous glands with CO_2_ laser (Sharplan 40 C, Lumenis, Yokneam, Israel) at 15–20 W in continuous wave mode.

At a comfortable working distance, with the laser handpiece held like a writing instrument for optimal control and a consistent tissue distance maintained by the pinky finger, the 300 mm focal length creates a 1.2 mm focal spot, allowing for both precise tissue ablation and coagulation.

Special attention was given throughout the procedure to visualizing the hypertrophic sebaceous glands as a ‘safe plane’ marker to avoid scarring. 

Following the procedure, patients were advised to practice sun avoidance, instructed on twice daily application of topical antibiotics (mupirocin) under dressing, and received prophylactic oral therapy with oral amoxicillin/clavulanic acid as well as acyclovir.

Patients were evaluated at 1-, 2-, 4- and 12-weeks following treatment.

Aesthetic outcome was assessed by the surgeon during the 3-month follow-up visit using a 0–5 scale (0–exacerbation, 1–0-24% improvement, 2–25-49% improvement, 3–50-74% improvement, 4–75-94% improvement and 5–95% improvement or greater).

### Long-term evaluation

To evaluate the long-term results of the treatment, patients were contacted by telephone and were asked to complete a questionnaire assessing satisfaction and recurrence. (Appendix 1)

## Results

Sixteen patients, 15 males and 1 female, are included in this report.

Average age at diagnosis was 67 years (range 29–89 years), and the average age at the time of treatment was 69 years (range 43–90).

Pre-procedural data were available for 13 patients (81%). The average duration of symptoms was 7.8 years (range 6 month-20 years).

While 2 patients (15%) also suffered from extra-nasal erythematotelangiectatic and papulopustular rosacea, none presented with phymatous changes in other locations.

Five patients were chronically prescribed with an anti-platelets drug (aspirin) and one with an anti-coagulant (rivaroxaban).

At the 3-month follow-up, 13 patients showed very good to excellent improvement.

Patient characteristics are described in Table 1.

### Questionnaire

Of the 16 patients, 13 (81%) completed the long-term follow-up questionnaire (2 patients passed-away from reasons unrelated to the procedure or to the rhinophyma and one was lost to follow-up) on an average of 15 months after the treatment (range 2–24 months).

Retrospectively grading pain throughout the procedure, 10 patients (77%) experienced no pain to mild pain, 2 reported moderate pain, and one reported severe pain.

Other adverse events were noted in only 2 patients: mild scarring in one and temporary swelling in the other.

All adverse events were short-term, required no medical intervention, and resolved within a few days. No long-term side effects, such as post-inflammatory hyperpigmentation or hypertrophic or keloid scars, were observed.

Eight patients (61%) reported that the aesthetic treatment outcome was fully maintained, 2 experienced minimal relapses, 2 had a partial relapse, and one experienced a complete recurrence 2.5 years following the procedure.

Of the eight patients who experienced increased sebum secretion prior to treatment, only 2 patients (25%) have suffered from secretions following the treatment.

Patient satisfaction following treatment was high, with an average satisfaction score of 7.9 out of 10 (range 4–10).

Eleven (85%) patients stated that they would recommend this treatment to someone with a similar problem.

Questionnaires’ data is presented in Table 2.

**Fig. 1:**
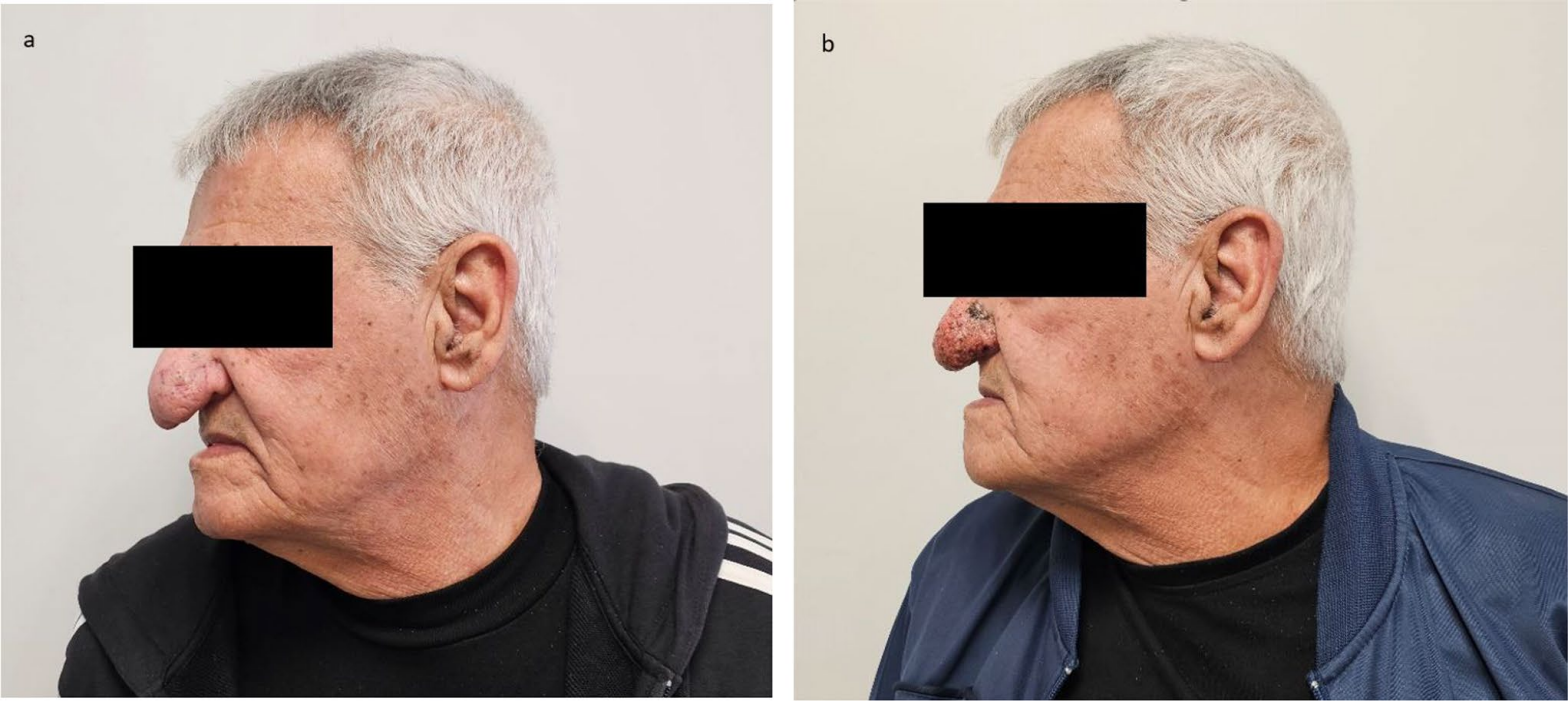
A 67-year-old patient (**a**) before treatment, displaying excessive lobular downsloping hypertrophy, and (**b**) one week after treatment, showing a visible reduction in nose size and a reshaped contour

**Fig. 2:**
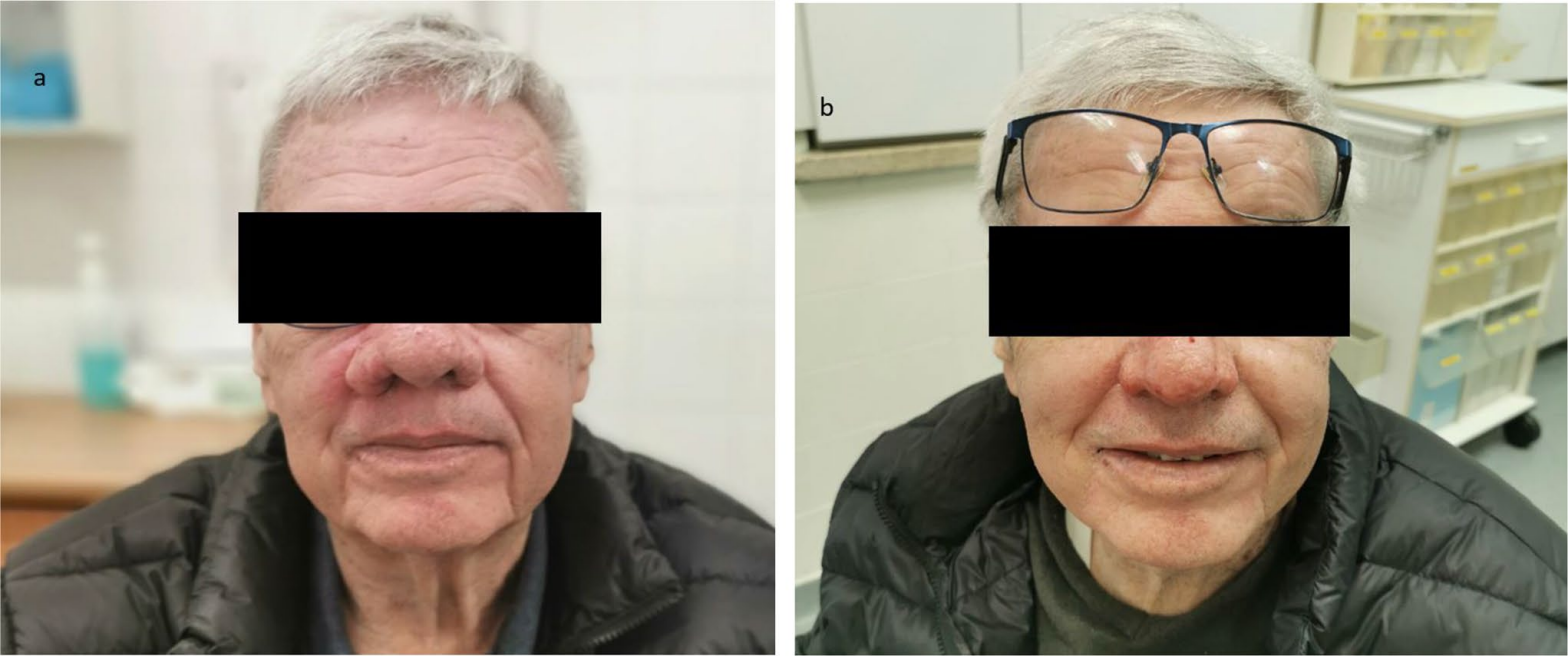
A 68-year-old (**a**) patient prior to treatment, exhibiting asymmetrical ala-nasi hypertrophy, and (**b**) one month following treatment with a significant improvement in the size and contour of the nose

Figures 1 and 2 display patients before (a) and after (b) treatment.

Supplementary video (Video 1) demonstrates phymateous tissue ablation to the level of the hypertrophied sebaceous glands that functions as a ‘safe plane’ marker, lowering the risk for scars.

**Table 1:** Demographic and clinical data of patients treated for rhinophyma with non-fractional ablative CO_2_ laser resurfacing

	Patients (*n* = 16)
Average age at diagnosis, y (range)	67 (29–89)
Average age at treatment, y (range)	69 (43–90)
Sex	
M (%)	15 (94%)
F (%)	1 (6%)
Aesthetic outcome evaluation^a^	
0 – exacerbation	0
1 – mild	0
2 – moderate	1
3 – good	2
4 – very good	4
5 – Excellent	9
	**Patients** (*n* **= 13)**
Average duration of symptoms, y (range)	7.8 (0.5–20)
Clinical presentation	
Increased sebaceous secretions	8
Extra-nasal rosacea	2
Itching	1
Prior treatment^b^	
Minocycline	5
Isotretinoin	2
Topical application	4

Abbreviations: y, year

^a^Aesthetic improvement was evaluated 3 months following the procedure on a scale of 0–5 -

0–exacerbation, 1–0-24% improvement, 2–25-49% improvement, 3–50-74% improvement,

4–75-94%, 5–95% improvement or greater

^b^Multiple treatments per patient were included

**Table 2:** Long term follow-up questionnaire results of patients treated for rhinophyma with non-fractional ablative CO_2_ laser resurfacing

	Patients (*n* = 13)
Reasons for treatment^a^	
Aesthetic	8
Increased sebum secretion	2
Swelling	2
Breathing difficulties	1
Persistent inflammation	1
Relapse	
None	8
Minimal	2
Partial	2
Complete	1
Sebaceous secretions following procedure	
No	11
Yes	2^b^
Pain during the procedure	
None to Mild (0–3)	10
Moderate (4–6)	2
Severe (7–10)	1
Adverse events	
None	11
Mild scarring	1
Swelling following procedure	1
Average satisfaction^c^	
Mild (4)	1
Moderate (5–7)	4
High (8–10)	8
Would recommend the procedure	
Yes	11
No	2

^a^Multiple reasons per patient were included

^b^Both experienced sebaceous secretions prior to treatment

^c^1 – ‘not satisfied at all’, 10 – ‘highly satisfied’

## Discussion

This study provides supporting evidence for the efficacy and safety of ablative CO_2_ laser resurfacing for the treatment of severe rhinophyma.

As previously reported in the literature, we also found a strong male predominance and occurrence peak by the 5th-7th decade of life [4]. 

Only 2 patients had also suffered from non-phymatous rosacea in extra-nasal areas. This finding further supports the notion that rhinophyma is not an end stage of erythematotelangiectatic or papulopustular rosacea, but rather a distinct subtype of it [15]. 

Performing the procedure under local anesthesia proved sufficient for alleviating pain during and short after the procedure as evident from the relatively low average over-all pain score (2/10). This is supported by other studies investigating the use of CO_2_ laser treatment for rhinophyma, including in a case of a massive rhinophyma, and is also consistent with the findings in other resurfacing indications [16–18]. This is a significant finding given the average age of rhinophyma patients, which makes general anesthesia less desirable [19]. 

Although 46% of patients were using antiplatelet or anticoagulant therapy, only mild bleeding was evident. This effect is primarily attributed to the hemostatic qualities of the ablative CO_2_ laser [20], but may also be owed in part to the pressure resulting from the infiltration of the local anesthetic.

Our findings of favorable and long-lasting results are clearly demonstrated by the 3-months evaluation results with and the high percentage of patients (85%) who state they would recommend ablative CO_2_ laser therapy to other rhinophyma patients. While this has been previously demonstrated in a larger questionnaire-based follow-up study involving 124 patients with minor to severe rhinophyma, the high participation rate in our study (81% compared to 42%) emphasizes our finding [12]. Additionally, our positive outcomes are particularly noteworthy given that, unlike in previous studies, all of our patient presented with severe rhinophyma.

This study has several limitations, primarily its retrospective nature, the relatively small number of patients and the lack of mild or moderate rhinophyma patients.

In conclusion, non-fractional ablative CO_2_ laser resurfacing performed under local anesthesia proved to be safe, well-tolerated, effective and sustainable treatment for rhinophyma with a high satisfaction rate.

## Electronic supplementary material

Below is the link to the electronic supplementary material.

Supplementary Material 1

Patient Questionnaire

## Data Availability

No datasets were generated or analysed during the current study.
