# Supplementary material for: Efficacy and safety of non-fractional ablative carbon dioxide laser resurfacing for the treatment of rhinophyma – a retrospective cohort and questionnaires-based study
Source: Lasers Med Sci. 2025 Jun 9;40(1):261. doi: 10.1007/s10103-025-04442-7 (PMC12146221; doi:10.1007/s10103-025-04442-7)
Supplement: Supplementary file 2 — Patient Questionnaire [file 10103_2025_4442_MOESM2_ESM.docx]

# Appendix 1 – Patient Questionnaire

1. What was your reason for undergoing the treatment (e.g., aesthetic concern, nasal discharge, other)?

________________________________________________________________________________

1. Were the treatment results sustained over time, or did any symptoms recur?

If symptoms did recur, please specify them (e.g., discharge, breathing difficulties, aesthetic concerns).

________________________________________________________________________________

1. Did you experience any pain during the laser treatment? (Yes/No)

________________

1. If you experienced pain during the laser treatment, what was the intensity of the pain? (0 – No pain, 10 – Unbearable pain)

________________

1. Did you experience any side effects following the treatment? (Yes/No)

If yes, please list them.

________________________________________________________________________________

1. Did you experience purulent discharge from the nasal skin before the treatment? (Yes/No)

________________

1. Did you experience purulent discharge from the nasal skin within one month after the treatment? (Yes/No)

________________

1. Did you experience purulent discharge from the nasal skin within three months after the treatment? (Yes/No)

_________________

1. Did you experience purulent discharge from the nasal skin within one year after the treatment? (Yes/No)

_________________

1. How satisfied are you with the treatment you received? (0 – Very dissatisfied, 10 – Very satisfied)

________________

1. Would you recommend this treatment to patients with similar symptoms? (Yes/No)

_______________
